# Development of a reliable UHPLC-MS/MS method for simultaneous determination of zearalenone and zearalenone-14-glucoside in various feed products

**DOI:** 10.3389/fchem.2022.955266

**Published:** 2022-08-10

**Authors:** Zhiqi Zhang, Yaling Cai, Kai Fan, Qingwen Huang, Xiuying Zhao, Haojie Cao, Zhihui Zhao, Emmanuel K. Tangni, Zheng Han

**Affiliations:** ^1^ Institute for Agro-Food Standards and Testing Technology, Shanghai Academy of Agricultural Sciences, Shanghai, China; ^2^ Organic Contaminants and Additives, Chemical and Physical Health Risks, Brussels, Belgium

**Keywords:** zearalenone, zearalenone-14-glucoside, ultra-high-performance liquid chromatography–tandem mass spectrometry, HLB cartridges, feed products

## Abstract

A reliable ultra-high-performance liquid chromatography–tandem mass spectrometry method (UHPLC-MS/MS) was developed for the simultaneous determination of two mycotoxins, that is, zearalenone (ZEN) and zearalenone-14-glucoside (ZEN-14G) in formula feed, concentrated feed, and premixed feed products. An improved sample pretreatment was achieved with the hydrophilic–lipophilic balance (HLB) cartridges efficiently removing the impurities and enriching the target analytes in different feeds. The critical parameters affecting the performance of the solid-phase extraction (SPE) procedure were carefully optimized, and 20% acetonitrile in water as the loading solution, 50% methanol in water as the washing solvent, and 5 ml of methanol as the elution solvent yielded the optimal purification efficiencies. The established method was thoroughly validated in terms of linearity (*R* ^2^ ≥ 0.999), sensitivity (limit of quantification in the range of 0.50–5.00 μg kg^−1^), recovery (89.35 ± 2.67% to 110.93 ± 1.56%), and precision (RSD, 3.00–14.20%), and it was then successfully applied to investigate a total of 60 feed samples. Among them, 50 samples were found to be contaminated with ZEN (an incidence of 83.3%) at levels ranging from 0.63 to 615.24 μg kg^−1^, whereas 22 samples were contaminated with ZEN-14G (an incidence of 36.7%) in the range of 0.89–15.31 μg kg^−1^. The developed method proved to be a specific and reliable tool for intensive monitoring of ZEN and ZEN-14G in complex feed matrices.

## Introduction

Zearalenone (ZEN), one of the most prevalent mycotoxins, is produced by *Fusarium* species and has attracted more and more attention due to safety concerns and economic impacts (Knutsen et al., 2017). As a liposoluble mycotoxin with a macrocyclic β-resorcyclic acid lactone, ZEN competes with endogenous hormones for the binding sites of estrogen receptors, leading to reproductive disorders in farm animals and hyperestrogenic syndromes in humans (Zinedine et al., 2007; Liu and Applegate, 2020). ZEN can easily contaminate various cereals during growth, harvest, or storage (Eskola et al., 2020). Based on a recent survey on the occurrence of ZEN, the frequently contaminated samples are maize, raw maize, wheat, beans, and feed mixtures, with the contamination rate of over 75% at a maximum concentration of 1,560 μg kg^−1^ (Ropejko and Twaruzek, 2021). The commonly used livestock animal feed ingredients and finished feeds were all highly contaminated with ZEN, that is, maize (44%), maize dried distiller grains with solubles (75%), wheat (33%), barley (20%), and finished feed (56%) (Gruber-Dorninger et al., 2019). The health risks and economic burdens posed by ZEN in feeds include both the intoxication of animals and the adverse effects on humans who eat contaminated animal-derived foods (Danicke and Winkler, 2015; Yang et al., 2020) and thus have become growing global concerns (Palumbo et al., 2020). The European Union (EU) has established guidance levels of 100–500 μg kg^−1^ for ZEN in complementary and complete feeding stuffs and 2,000–3,000 μg kg^−1^ in feed materials (Commission of the European Communities, 2006).

Recently, masked mycotoxins, generated from the defense mechanism of plants against xenobiotics, have emerged as important contaminants because of their unpredicted toxicities and hard-to-detect tendencies (He et al., 2018; Zhang et al., 2020). The most frequently discussed masked form of ZEN is its glucose conjugate zearalenone-14-glucoside (ZEN-14G) (Binder et al., 2017; Dellafiora et al., 2017; Lorenz et al., 2019). ZEN-14G was found in wheat at levels ranging from 17 to 104 μg kg^−1^ and is highly correlated to ZEN with a 42% co-occurrence rate (Isabell et al., 2002). ZEN-14G was also detected in maize with large amounts of 274 μg kg^−1^ (Boevre et al., 2012a) and 199 μg kg^−1^ (Boevre et al., 2012b). Although ZEN-14G exhibits lower toxicity than its original form (Cirlini et al., 2016; Dellafiora et al., 2016), the conversion of ZEN-14G to ZEN by intestinal flora during digestion may pose an additional risk to human and animal health (Dall'Erta et al., 2013; Gratz et al., 2017). Currently, ZEN-14G is not considered in any regulation or EU guidance (Knutsen et al., 2017), and thus, it is frequently not involved in routine analysis, resulting in a significant underestimation of the actual harmful effects. As a consequence, it is a critical issue to establish efficient analytical methods for the accurate detection of not only ZEN but also its masked form, ZEN-14G, in feeds.

Various analytical methods, for example, thin-layer chromatography (TLC), enzyme-linked immunosorbent assay (ELISA), liquid chromatography coupled with mass spectrometry (LC-MS) or a fluorescence detector (FLD), gas chromatography (GC) –MS, and high-performance liquid chromatography–tandem mass spectrometry (HPLC-MS/MS), have been established for detecting and quantifying a variety of mycotoxins in cereals and cereal products (EFSA Panel on Contaminants in the Food Chain (CONTAM), 2016; Munoz-Solano and Gonzalez-Penas, 2020; Pereira et al., 2014; Santos et al., 2019). TLC is simple and fast, but the low sensitivity and unsatisfying accuracy limit its application. ELISA methods focus on the screening purpose, and the positive findings should be confirmed by other analytical techniques (Ran et al., 2013). Among these techniques, the most promising tool is HPLC-MS/MS because of its universality, high accuracy, and sensitivity (Romera et al., 2018), and it has been utilized to determine ZEN and ZEN-14G in cereals and cereal-based foods (Beloglazova et al., 2013; De et al., 2013; Chilaka et al., 2019; Rausch et al., 2020). However, the complex constituents (lipids, fatty acids, proteins, and other components) that could severely interfere with the separation and ionization process make these methods inadequate in feed products (Romera et al., 2018). Because of the completely different polarities of ZEN and ZEN-14G, to the best of our knowledge, few methods have been established for simultaneous analysis of ZEN and ZEN-14G in different feeds.

To face these analytical challenges, the aim of our study was to develop a reliable and sensitive ultra-HPLC-MS/MS (UHPLC-MS/MS) method for the simultaneous determination of ZEN and ZEN-14G in various feeds. The established analytical method based on an efficient hydrophilic–lipophilic balance–solid-phase extraction (HLB-SPE) clean-up approach was extensively validated by determining the linearity, sensitivity, recovery, and precision and then applied for monitoring the two analytes in different feed samples to reveal the real contamination situation in China.

## Materials and methods

### Reagents and chemicals

The ZEN and ZEN-14G standards were obtained from Sigma-Aldrich (St. Louis, MO, USA) and HPC standards GmbH (Borsdorf, Germany), respectively (Supplementary Table S1). C_18_ and HLB-SPE cartridges were acquired from NanoChrom Co. (Suzhou, China). PriboFast^®^MFC 260 cartridges were purchased from Pribolab (Qingdao, China). Methanol and acetonitrile were purchased from Merck (Darmstadt, Germany). Water used throughout the whole analysis was Milli-Q quality water (Millipore, Billerica, MA, United States). All reagents and chemicals were of analytical or HPLC grade.

### Samples

A total of 60 feed samples were randomly collected from different formal enterprises in Shanghai, Jiangxi province, and Guangdong province in China, consisting of three types: formula feed products (28), concentrated feed products (14), and premixed feed products (18). It was verified that neither ZEN nor ZEN-14G was detected in samples used as blank matrices. All samples were finely ground using a high-speed grinder (Supor Co., Ltd., Zhejiang, China), passed through a 0.45 mm sieve, and stored at 4°C until analysis.

### Sample pretreatment

Each sample (2.0 g) was accurately weighed into a 15 ml centrifuge tube and was macerated with 10 ml of acetonitrile/water/formic acid (79:20:1, v/v/v) for 5 min, followed by ultrasonic extraction for 30 min at 40°C (Shanghai Anpu Co., Ltd., Shanghai, China). After centrifugation at 13,000 *g* for 5 min, 1 ml of the supernatant was collected and dried with nitrogen gas (Organomation Associates, Inc. Berlin, MA, United States) at 40°C. The residues were redissolved in 5 ml of acetonitrile/water (20:80 v/v) and passed through the HLB-SPE cartridges, which were pre-equilibrated with 2 ml of methanol and acetonitrile/water (20:80,v/v) at a flow rate of 1–2 drops/s successively. Then, the cartridges were washed with 5 ml of methanol/water (50:50, v/v). All target analytes were eluted with 5 ml of methanol, dried with nitrogen gas at 40°C, and redissolved in 1 ml of acetonitrile/water containing 5 mmol L^−1^ ammonium acetate (70/30, v/v). Finally, the eluent was passed through a 0.22 μm nylon filter (Pall, Port Washington, NY, United States), ready for UHPLC-MS/MS analysis.

### UHPLC-MS/MS analysis

UHPLC analysis was performed to separate ZEN and ZEN-14G *via* a Waters ACQUITY HPLC system (Waters, Milford, MA, United States) at 40 °C using a Poroshell 120 EC-C_18_ column (2.1 mm × 100 mm, 1.9 μm) (Agilent, Santa Clara, CA, United States). A linear gradient elution was carried out at a flow rate of 0.3 ml min^−1^ using (A) methanol and (B) 5 mmol L^−1^ ammonium acetate aqueous solution as the mobile phase, and the gradient program was designed as follows: 0.0–1.0 min 10% A, 1.0–3.0 min 10–25% A, 3.0–6.0 min 25% A, 6.0–7.0 min 25–90% A, 7.0–7.5 min 90% A, 7.5–7.6 min 90–10% A, and 7.6–9.0 min 10% A. The injection volume was 3 μL.

MS/MS analysis was performed on a Triple-Quad™ 5,500 mass spectrometer (AB Sciex, Foster City, CA, USA) equipped with an electrospray ionization (ESI) interface operating in the positive mode (ESI^+^) for ZEN-14G and the negative mode (ESI^−^) for ZEN. The parameters were set as follows: ion spray voltage, 5.5 kV (ESI^+^) and 4.5 kV (ESI^−^); block source temperature, 500°C; nebulizer gas, 50 psi; turbo gas pressures, 50 psi; curtain gas pressure, 35 psi; and collision gas value, medium. Multiple reaction monitoring (MRM) was performed for the quantification of ZEN and ZEN-14G (Table 1). Data acquisition and processing were performed with the MultiQuant algorithm from MultiQuant 3.0.2 (Analyst; AB SCIEX).

**TABLE 1:** MS/MS parameters of ZEN and ZEN-14G.

Mycotoxins	Retention Time (min)	Precursor Ion (*m/z*)	Products Ion (*m/z*)	Collision Energy (eV)
ZEN	8.00	316.90	174.90^a^	−35
			131.00	−40
ZEN-14G	7.75	481.20	319.20^a^	12
			283.10	26

a Primary product ions.

### Method validation

The performance of the established method was thoroughly validated by determining the selectivity, linearity, limit of detection (LOD), limit of quantification (LOQ), recovery, and precision for each matrix (formula feed, concentrated feed, and premixed feed). The selectivity of the method was investigated by comparing the MRM chromatograms of the uncontaminated feed samples, contaminated feed samples, blank matrices spiked with ZEN and ZEN-14G (50 ng ml^−1^), and the standard solutions. A series of standard solutions for ZEN and ZEN-14G with concentrations of 0.05, 0.1, 0.2, 0.5, 1, 2, 5, 10, 20, 50, 100, and 200 ng ml^−1^ were prepared in neat solvents (acetonitrile/water containing 5 mmol L^−1^ ammonium acetate, 70/30, v/v) and blank matrices. Linear calibration curves (1/*x* weighted) were obtained for ZEN and ZEN-14G by plotting MS responses (peak areas) versus the concentrations of the analytes. The sensitivity of the method was evaluated by the determination of LOD and LOQ, defined as the concentrations of the analytes generating signal-to-noise (*S*/*N*) ratios of 3 and 10 in each matrix, respectively. Blank samples spiked with 1, 5, 20, 50, and 100 μg kg^−1^ for ZEN and 5, 10, 20, 50, and 100 μg kg^−1^ for ZEN-14G were utilized to investigate the recovery, intra-day, and inter-day precisions (*n* = 5). The recovery of the overall process efficiency (*PE*) was calculated by comparing the concentration levels measured by the matrix-matched calibration curves with the spiked levels according to the following formula (Purohit et al., 2020). Recovery values between 70 and 120% were considered to be acceptable. Repeatability, expressed as the relative standard deviations (RSDs), was determined as the variations within the same day for the intra-day precision and over 5 consecutive days for the inter-day precision.

$${\text{Recovery}}_{\text{PE}}=\text{Measured concentration}/\text{Nominal concentration}\times 100\%$$ (1)

### Matrix effect

The matrix effect (ME) was evaluated by determining the signal suppression/enhancement (SSE) values, calculated by comparing the slope of the matrix-matched calibration curves with that constructed in the neat solvent according to the following equation (Tanveer et al., 2020):

$$\mathrm{ME}={\mathrm{Slop}e_{\mathrm{matrix spiked}}/\mathrm{Slope}}_{\mathrm{standard solution}}\times 100\%$$ (2)

### Statistical analysis

Data analyses were performed using the software SPSS Statistics 19 (SPSS Inc., Chicago, IL, USA). The results were described by means ± standard deviations ( $\bar{X}$ ± SD) and RSDs.

## Results and discussion

### Optimization of UHPLC conditions

To obtain optimal separation and ionization efficiencies, different mobile phases were compared: 1) methanol–water, 2) methanol–water containing 0.1% formic acid, 3) methanol–water containing 5 mmol L^−1^ ammonium acetate, and 4) methanol–water containing 0.1% formic acid and 5 mmol L^−1^ ammonium acetate. The results of multiple injections indicated that the responses of ZEN and ZEN-14G were greatly improved and the highest sensitivity was obtained when methanol–water containing 5 mmol L^−1^ ammonium acetate was selected. Under such a situation, a satisfactory separation efficiency and a nice peak shape were also achieved (Supplementary Figure S1). Subsequently, the efficiencies of different concentrations of ammonium acetate (methanol–water containing 3, 5, and 7 mmol L^−1^ ammonium acetate) were further investigated (Supplementary Figure S2). We found that the effects of 3 mmol L^−1^ ammonium acetate and 5 mmol L^−1^ ammonium acetate were comparable, and both were better than 7 mmol L^−1^ ammonium acetate. Alternatively, methanol–water containing 5 mmol L^−1^ ammonium acetate was selected due to its relatively higher sensitivity for ZEN.

### Optimization of sample preparation

#### Sample extraction

**FIGURE 1:**
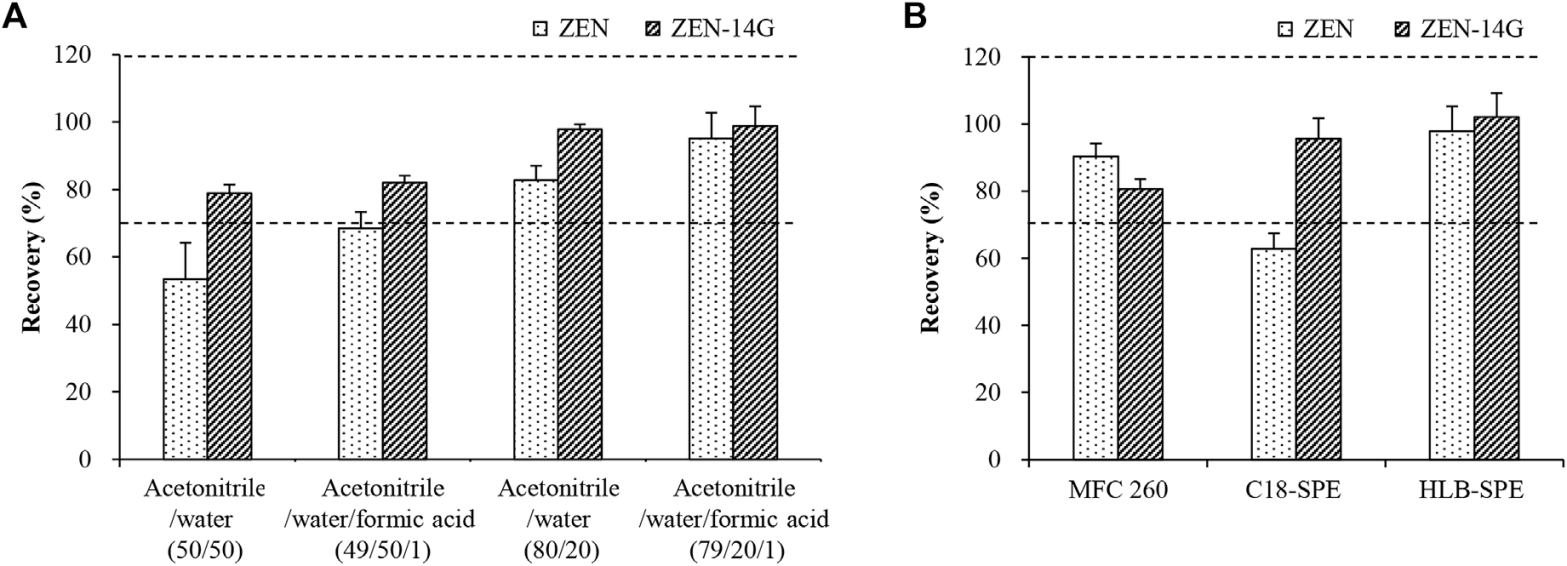
Comparison of the extraction efficiencies for ZEN and ZEN-14G using the four candidate extraction solvents **(A)** and purification efficiencies by three different clean-up cartridges **(B)** using spiked formula feed samples (50 μg kg^−1^). Data are presented as the mean ± standard deviation (SD). Acceptable recoveries are in the range of the two dashed lines (70–120%).

Extraction efficiencies were evaluated by the recovery of the extraction process (*RE*) according to the following formula using the spiked formula feed samples (50 μg kg^−1^) (Caban et al., 2012). First, the extraction efficiencies of three commonly used organic solvents (methanol, acetone, and acetonitrile) were compared. Among the three solvents, acetonitrile had the highest extraction efficiency, but it still failed to achieve the desired recovery (Supplementary Figure S3). Then, four differently composed solvents including (i) acetonitrile/water (50/50, v/v), (ii) acetonitrile/water/formic acid (49/50/1, v/v/v), (iii) acetonitrile/water (80/20, v/v), and (iv) acetonitrile/water/formic acid (79/20/1, v/v/v) were investigated. Higher water contents in the extraction solvents (i and ii) were drastically unsuitable for ZEN (Figure 1A). The neutral solvent (iii) and acidic solvent (iv) with lower water contents gave better results for ZEN (iii/82.7 ± 4.3%; iv/95.1 ± 7.6%) and ZEN-14G (iii/97.8 ± 1.5%; iv/98.8 ± 5.9%), respectively. This was consistent with the previous studies that extraction solvents with high water contents led to low recoveries for ZEN and its derivatives (Vendl et al., 2009; Boevre et al., 2012b). Alternatively, the acidic solvent (iv) was selected due to the relatively higher recoveries for both the analytes. This may be related to the fact that an acidic medium facilitates the extraction of mycotoxins into organic solvents (Munoz-Solano and Gonzalez-Penas, 2020). Then, the extraction efficiencies of different percentages of formic acid (0.5, 0.75, 1, 2, and 4%) were further compared. The results indicated that 1% formic acid worked the best (Supplementary Figure S4). Finally, acetonitrile/water/formic acid (79/20/1, v/v/v) was selected for the subsequent experiments.

RE=A/B×100% (3)

A denotes the peak area of the analyte recorded for the sample spiked with the target compound(s) before extraction, and B denotes the peak area of the analyte recorded for the sample spiked with the target compound(s) after extraction.

#### Optimization of the SPE clean-up procedure

Molecules originating from the samples that were coextracted with the analytes of interest might interfere with the ionization process in the mass spectrometer, resulting in ionization SSE. Different clean-up cartridges (PriboFast^®^MFC 260, C_18_-SPE, and HLB-SPE) were compared using the spiked formula feed samples (50 μg kg^−1^). As shown in Figure 1B, for MFC 260, only ZEN gave an acceptable recovery of 90.3 ± 3.9%, whereas unsatisfactory recovery was obtained for the polar analyte ZEN-14G. In contrast, when C_18_-SPE cartridges were used, only ZEN-14G achieved a satisfactory recovery. With respect to HLB-SPE cartridges, satisfactory recoveries of 97.84 ± 7.36% for ZEN and 102.11 ± 7.14% for ZEN-14G were reached. This may be attributed to the fact that HLB cartridges contain apolar and polar regions (Jeong et al., 2017), allowing a good ability to interact with both the apolar and polar molecules, and thus are suitable to accommodate both ZEN and ZEN-14G.

#### Acetonitrile content in the loading solutions

**FIGURE 2:**
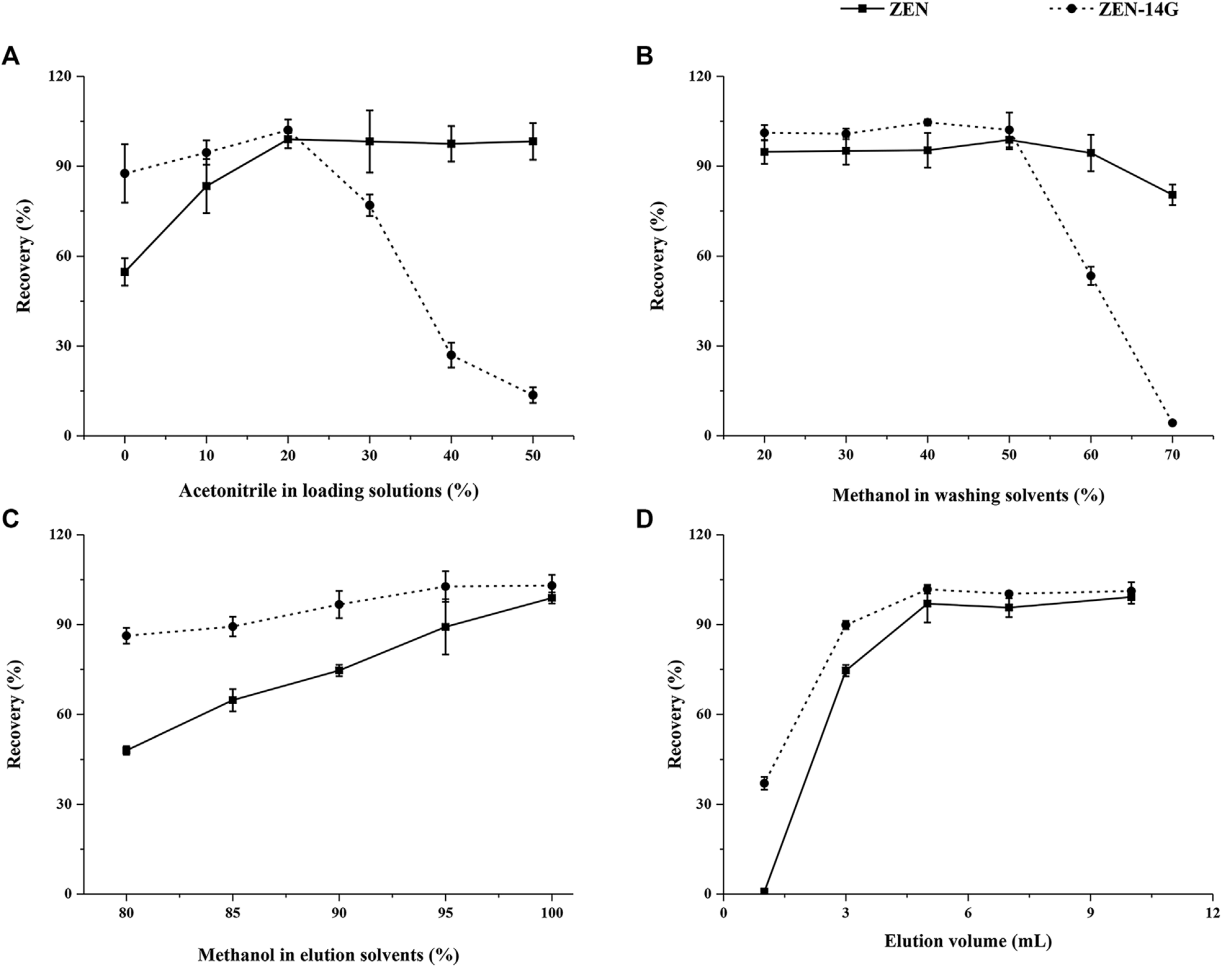
Optimization of the critical parameters on the performance of the HLB-SPE cartridges including the percentage of acetonitrile in sample loading solutions **(A)**, percentage of methanol in washing solvents **(B)**, percentage of methanol in elution solvents **(C)**, and elution volume **(D)** using blank formula feed samples spiked with 50 μg kg^−1^ for both analytes (n = 5).

In general, the proportion of organic solvents in the loading solutions plays a critical role in the adsorption efficiency of HLB-SPE. To improve the purification efficiency, different percentages of acetonitrile (0, 10, 20, 40, and 50%) were compared (Jiang et al., 2018). With the increasing percentages of acetonitrile, the recoveries of ZEN increased dramatically and then showed a steady trend, whereas ZEN-14G increased only slightly at first and then dropped sharply. Satisfactory recoveries for both ZEN and ZEN-14G were achieved when water containing 20% acetonitrile was used as the adsorption solution (Figure 2A).

#### Optimization of the washing solvents

To reduce the presence of possible interference caused by organic compounds in feed matrices, such as lipids, proteins, and carbohydrates, different methanol–water solutions as the washing solvents, 1) 20/80 (v/v), 2) 30/70 (v/v), 3) 40/60 (v/v), 4) 50/50 (v/v), 5) 60/40 (v/v), and 6) 70/30 (v/v), were tested (Chen et al., 2012). As shown in Figure 2B, with the increasing percentages of methanol in the washing solvents, the recoveries of ZEN and ZEN-14G showed steady trends at first. Then, ZEN showed a slightly declined tendency, whereas ZEN-14G dropped sharply. When the organic phase proportion condition (50% methanol) was chosen, ideal recoveries were obtained for ZEN and ZEN-14G. We also found that the same proportion of acetonitrile showed a very different effect, especially for ZEN-14G; the recovery was almost zero (Supplementary Figure S5). Therefore, acetonitrile was not suitable as a washing solvent to remove impurities due to its higher polarity (Rubert et al., 2012).

#### Comparison of different elution solvents

A total of five different elution solvents, that is, (A) methanol–water (80/20, v/v), (B) methanol–water (85/15, v/v), (C) methanol–water (90/10, v/v), (D) methanol–water (95/5, v/v), and (E) methanol, were compared (Tanveer et al., 2020). The elution abilities of the solutions dramatically improved with the increasing proportions of methanol until it achieved 100%, leading to the highest recoveries for ZEN. As for ZEN-14G, the elution abilities evidently improved with increasing proportions of methanol until 90% and then remained relatively steady to 100% (Figure 2C). In addition, considering that pH value might affect elution efficiency (Wen et al., 2020), the influences of 1% formic acid or 1% ammonia in methanol were also investigated (Supplementary Figure S6), and the highest efficiencies were observed for the natural solvent. As a consequence, methanol seemed to be the best choice for the purification of the two analytes.

The elution volumes from 1 to 10 ml were also studied. As shown in Figure 2D, when the volumes increased from 1 to 5 ml, the recoveries of ZEN dramatically improved, whereas they showed a gentle increase for ZEN-14G. Then, when the volumes increased from 5 to 10 ml, the recoveries remained almost constant ranging from 97.0 ± 6.3% to 99.2 ± 2.2% and from 98.9 ± 2.0% to 101.2 ± 3.0% for ZEN and ZEN-14G, respectively. Therefore, 5 ml of methanol was selected to constitute a convenient and effective procedure.

#### Characterization of the HLB-SPE clean-up method

**FIGURE 3:**
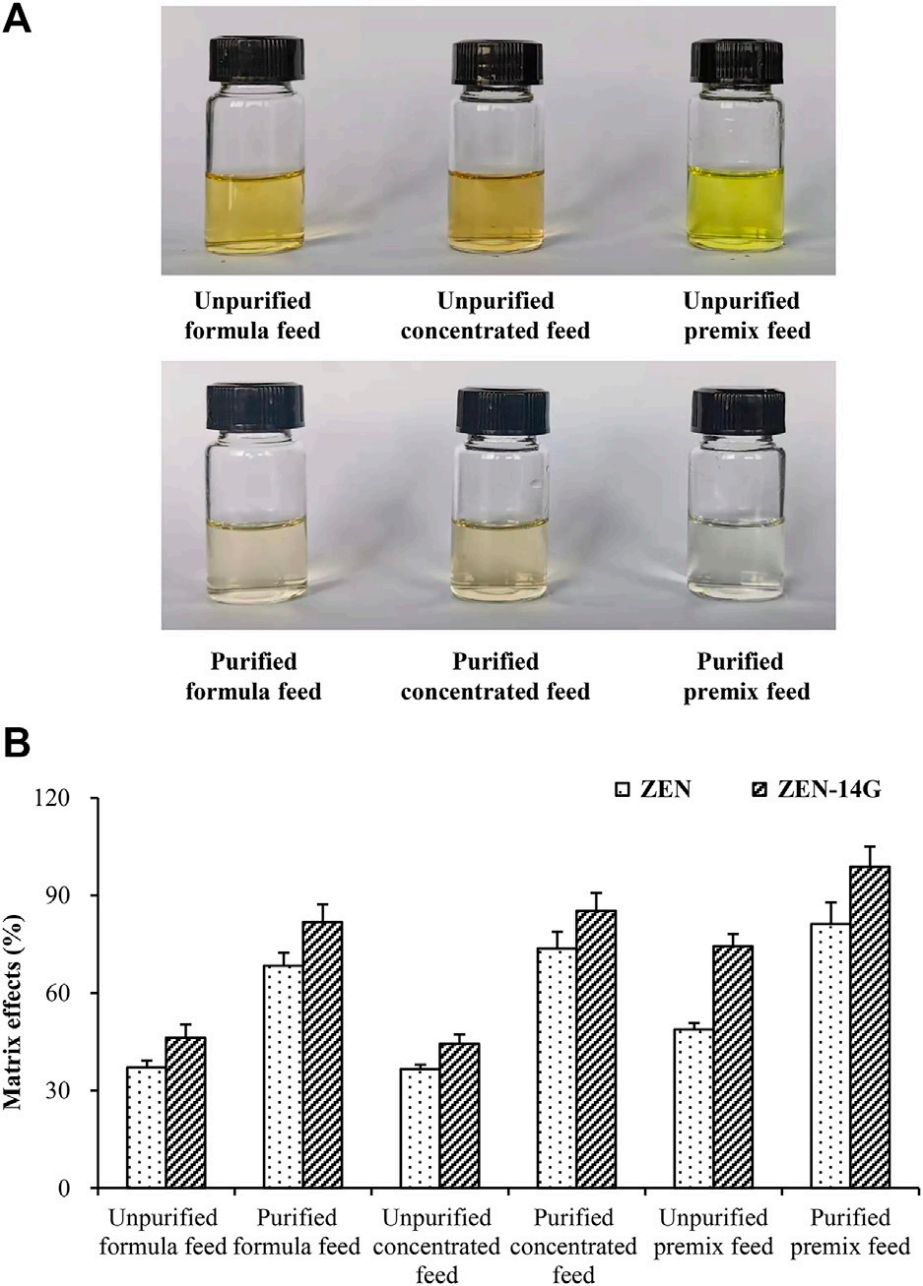
Visual appearance features **(A)** and matrix effects **(B)** of ZEN and ZEN-14G in various feeds purified or unpurified by the HLB-SPE cartridges.

To characterize and clarify the established clean-up method, the visual appearance characteristics and MEs of ZEN and ZEN-14G in different feed matrices, purified or unpurified with the HLB-SPE cartridges, were compared. As shown in Figure 3A, the solutions purified with the HLB-SPE cartridges were apparently lighter and transparent, demonstrating that the established method could efficiently remove the impurities from the feed matrices to minimize the interferences during MS/MS detection. The signal suppression values for ZEN and ZEN-14G of the unpurified samples were in the range of 37.1 ± 2.1% to 48.8 ± 1.9% and 44.4 ± 2.8% to 74.4 ± 3.7%, respectively (Figure 3B). After clean-up, the MEs of the two target analytes in different feed matrices were both clearly reduced. With regard to ZEN-14G, satisfactory signal suppression values of 81.8 ± 5.4%, 85.2 ± 5.5%, and 98.7 ± 6.2% were obtained for the formula feed, concentrated feed, and premixed feed matrix, respectively. This suggested that after clean-up with HLB cartridges, there were minimal matrix interferences in detecting ZEN-14G. Nevertheless, unsatisfactory values for ZEN were obtained in the feed matrices except for premixed feeds (81.2 ± 6.6%). Hence, matrix-matched calibration curves were constructed to guarantee accurate results.

### Method validation

**FIGURE 4:**
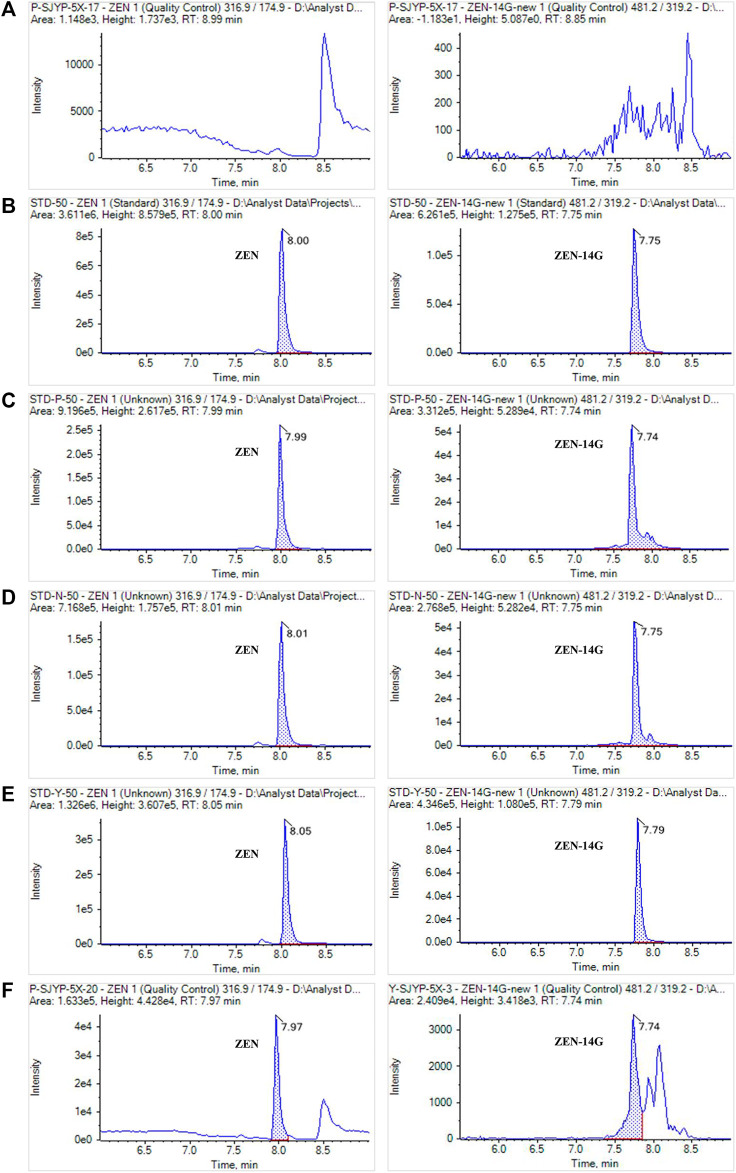
MRM chromatograms of ZEN and ZEN-14G in a blank feed sample **(A)**, in the neat solvent standard solution **(B)**, in the formula feed matrix standard solution **(C)**, in the concentrated feed matrix standard solution **(D)**, in the premixed feed matrix standard solution **(E)**, and in real contaminated feed samples **(F)**. The concentrations of standard solutions were 50 ng ml^−1^ for ZEN and ZEN-14G.

As previously reported, the successful application of SPE and the UHPLC-MS/MS method verified its advantage in analyzing the low level of contaminates (Chen et al., 2012; Jin et al., 2017). The method was selective for the detection of ZEN and ZEN-14G in different feeds because no interference peaks appeared at the retention time of these two analytes in the blank samples, also indicating that no ZEN and ZEN-14G existed in the selected blank matrix (Figure 4). Good linear relationships with coefficients of determination (*R* ^2^) higher than 0.999 were obtained for ZEN and ZEN-14G in the neat solvent and in different feed matrices (Table 2, Supplementary Figure S7). The LOD and LOQ values for ZEN and ZEN-14G were in the range of 0.15–2.00 μg kg^−1^ and 0.50–5.00 μg kg^−1^, respectively, proving the high sensitivity of the established method. There is little evidence to demonstrate the LOD and LOQ of ZEN-14G in the feed. The LOD values of ZEN-14G here were lower than 7.0 μg kg^−1^ reported by Beloglazova et al. in the feed (Beloglazova et al., 2013). The LOQ values were in line with that obtained by Rausch et al. (Rausch et al., 2020) but lower than the results reported by Chilaka et al. (Chilaka et al., 2019) in cereals and cereal-based foods. As for ZEN, the LOD and LOQ were comparable to the values obtained by Majer–Baranyi et al. (Majer-Baranyi et al., 2021) but lower than the results reported by Munoz–Solano and Gonzalez–Penas in the feed (Munoz-Solano and Gonzalez-Penas, 2020). Adequate *PE* recoveries of the two target analytes with the mean values in the ranges of 93.06 ± 1.42% to 110.93 ± 1.56% for the formula feeds, 89.35 ± 2.67% to 103.77 ± 2.66% for the concentrated feeds, and 93.46 ± 1.47% to 106.86 ± 5.29% for the premixed feeds were obtained (Table 3). The RSD values of ZEN and ZEN-14G were in the range of 3.00–7.96% for intra-day precision and 3.78–14.20% for inter-day precision, respectively. Based on the abovementioned validation details, the established UHPLC-MS/MS method was selective, sensitive, accurate, and repeatable and could fully meet the needs for routine monitoring of ZEN and ZEN-14G in different feed samples.

**TABLE 2:** Calibration curves and sensitivities of ZEN and ZEN-14G in the neat solvent and in various feed matrices.

Matrix	Mycotoxins	Linear Range (ng mL^−1^)	Slope	Intercept	*R* ^2^	LOD^a^	LOQ^b^
						(μg kg^−1^)	(μg kg^−1^)
Neat solvents	ZEN	0.10–200	42,994 ± 2,578	4,680 ± 391	0.999	-	-
	ZEN-14G	0.20–200	11,189 ± 429	892 ± 44	0.999	-	-
Formula feed	ZEN	1.00–200	30,472 ± 1,137	239,845 ± 23,024	0.999	0.30	1.00
	ZEN-14G	5.00–200	9,153 ± 604	196 ± 20	0.999	2.00	5.00
Concentrated feed	ZEN	0.50–200	31,676 ± 2,201	70,112 ± 3,329	0.999	0.15	0.50
	ZEN-14G	5.00–200	9,356 ± 557	1,320 ± 144	0.999	2.00	5.00
Premixed feed	ZEN	0.50–200	34,899 ± 2,854	9,259 ± 349	0.999	0.15	0.50
	ZEN-14G	0.50–200	11,048 ± 689	1,212 ± 88	0.999	0.20	0.50

**TABLE 3:** Recoveries, intra-day, and inter-day precisions of ZEN and ZEN-14G in various feed matrices (%, *n* = 5).

Mycotoxin Levels (μg kg^−1^)	Spiked Levels (μg kg^−1^)	Formula Feed			Concentrated Feed			Premixed Feed		
		Recovery	Intra-RSD	Inter-RSD	Recovery	Intra-RSD	Inter-RSD	Recovery	Intra-RSD	Inter-RS
		(±SD)			(±SD)			(±SD)		
ZEN	1	98.71 ± 9.49	4.79	8.23	94.46 ± 7.22	3.00	3.78	96.83 ± 6.39	5.89	9.99
	5	93.32 ± 4.24	4.64	7.28	89.35 ± 2.67	7.19	14.20	100.06 ± 2.40	5.41	5.46
	20	95.39 ± 3.14	4.59	11.29	94.62 ± 6.50	7.89	10.88	99.94 ± 4.27	7.04	10.25
	50	97.84 ± 7.36	5.95	6.22	93.36 ± 3.99	4.04	6.93	93.46 ± 1.47	5.17	10.47
	100	93.06 ± 1.42	3.06	3.81	94.39 ± 6.00	3.09	6.10	97.87 ± 1.87	3.86	7.72
ZEN-14G	5	99.83 ± 8.09	7.43	10.41	103.67 ± 2.70	7.96	11.25	102.63 ± 4.06	4.64	5.75
	10	110.93 ± 1.56	5.94	10.48	103.62 ± 6.76	6.81	8.36	95.30 ± 4.71	4.19	8.48
	20	102.65 ± 7.98	7.29	13.00	103.77 ± 2.66	7.21	10.56	100.63 ± 6.23	4.72	8.44
	50	102.11 ± 7.14	4.85	5.96	102.00 ± 2.60	7.77	12.83	103.93 ± 2.82	4.91	6.94
	100	99.87 ± 3.96	3.72	9.39	100.83 ± 4.57	3.83	6.13	106.86 ± 5.29	3.51	4.98

### Method application

Feed sources are extraordinarily diverse, and their compositions vary with not only the target species but also the age. A total of 60 feed samples for pigs (50) and poultry (10) at different growth stages, namely, 28 formula feeds, 14 concentrated feeds, and 18 premixed feeds, were collected and analyzed by the established UHPLC-MS/MS method (Table 4; Supplementary Table S2). Among the 60 samples, 50 were contaminated with ZEN with concentrations ranging from 0.63 to 615.24 μg kg^−1^. The incidence of ZEN in the current study was much higher than that (56%) reported in a global mycotoxin survey from 19,171 samples of 100 countries over 10 years (Gruber-Dorninger et al., 2019). However, the contamination rate of ZEN was lower than the previous studies in Slovakia (88%, range: 3–86 μg kg^−1^; mean value: 21 μg kg^−1^) (Labuda et al., 2005), Poland (90%, mean value: 21.7 μg kg^−1^) (Kosicki et al., 2016), South Korea (96.4%, range: 1–932 μg kg^−1^; mean value: 70 μg kg^−1^) (Chang et al., 2017), and China (96.9–100%, mean value: 48.1–326.8 μg kg^−1^) (Zhao et al., 2021). Differences in the occurrence of ZEN among various geographical areas may be attributed to the variances of the seasonal and local weather conditions during plant growth stages and the sample collection periods. As for ZEN-14G, very low levels ranging from 0.89 to 15.31 μg kg^−1^ were detected (an incidence of 36.7%).

**TABLE 4:** Occurrence of ZEN and ZEN-14G in formula feed, concentrated feed, and premixed feed samples.

Mycotoxins	Formula Feed		Concentrated Feed		Premixed Feed	
	Positive/Total Samples	Range (μg kg^–1^)	Positive/Total Samples	Range (μg kg^–1^)	Positive/Total Samples	Range (μg kg^−1^)
ZEN	23/28	1.71–615.24	11/14	0.63–62.30	16/18	3.00–194.22
ZEN-14G	8/28	6.12–8.85	4/14	5.28–9.26	10/18	0.89–15.31

There were few differences in the incidence of ZEN among the formula feeds (82.1%), concentrate feeds (78.6%), and premixed feeds (88.9%). Poultry feeds were contaminated with ZEN in the range of 4.0–615.2 μg kg^−1^ with a positive rate of 90.0%, but only two samples were contaminated with ZEN-14G at the levels of 1.8 μg kg^−1^ and 2.5 μg kg^−1^. Only one formula feed sample contained ZEN (615.2 μg kg^−1^) with a concentration higher than the limit established by China (500 μg kg^−1^) (Supplementary Table S3). The contamination levels of ZEN in the poultry feed samples in our study were higher than the results reported in Nigeria (83.8%, range: 0.5–71 μg kg^−1^; mean value: 9.3 μg kg^−1^) (Akinmusire et al., 2019) but lower than that reported in South Korea (96.3%, mean value: 37.9 μg kg^−1^) (Chang et al., 2017), Kenya (100.0%, range: 5.2–873.4 μg kg^−1^) (Kemboi et al., 2020), and South Africa (100%, range: 0.1–429.0 μg kg^−1^) (Mokubedi et al., 2019). Feeds for pigs appeared to be contaminated with ZEN at a positive rate of 82.0% in the range of 0.6–194.2 μg kg^−1^, whereas the positive rate for ZEN-14G was 40.0% with a maximum value of 15.3 μg kg^−1^. The contamination by ZEN in pig feeds was higher than the results obtained by Thieu et al. (2008) in southern Vietnam (67.0%, range: 10.0–571.0 μg kg^−1^) (Thieu et al., 2008) but lower than the results reported by Chang et al. (2017) in South Korea (95%, mean value: 31.7 μg kg^−1^) (Chang et al., 2017). In a recent study on the occurrence of multimycotoxin occurrence between 2018 and 2020 in China, ZEN was found in 99.6–100% of poultry feed samples at the mean concentrations of 59.8–155.1 μg kg^−1^ (Zhao et al., 2021). In the same study, the incidences and concentrations in pig feeds were 98.4–100% and 67.2–93.1 μg kg^−1^, respectively. A similar occurrence pattern was observed in another previous investigation conducted in China, in which the occurrence rate of ZEN was more than 99.0 and 10.8% exceeding China’s regulatory limits (Ma et al., 2018). Compared with the two studies conducted in China, obviously light contamination situations were observed in the current work with ZEN concentrations in all samples within the limits set by the EU and China for pigs (100–250 μg kg^−1^) (Supplementary Table S3).

It is worth noting that ZEN co-occurred with ZEN-14G in 21 feed samples (an incidence of 35.0%) with concentrations of 0.71–119.66 μg kg^−1^ and 0.89–15.31 μg kg^−1^, respectively. Although there are no recommendations or regulations on ZEN-14G in feeds, the occurrence of ZEN-14G would certainly increase the adverse effects on animal and human health (Dellafiora et al., 2016; Yang et al., 2018; Lorenz et al., 2019).

## Conclusion

A sensitive and reliable UHPLC-MS/MS method based on the commercially available HLB-SPE cartridges was established for the simultaneous determination of ZEN and its masked form, ZEN-14G, in formula feed, concentrated feed, and premixed feed products. After the optimization of the SPE procedure and UHPLC-MS/MS conditions, the established method was demonstrated to be rapid, sensitive, effective, accurate, and efficient to monitor ZEN and ZEN-14G in different feed samples, which also shows its feasibility for broader applications in the field of analytical chemistry. Considering the highly toxic characteristics of ZEN alongside its high co-occurrence with the masked form, continuous monitoring in various feeds using the developed UHPLC-MS/MS method is recommended.

## Data Availability

The original contributions presented in the study are included in the article/Supplementary Material. Further inquiries can be directed to the corresponding author.

## References

[B1] Akinmusire O. O. El-Yuguda A. D. Musa J. A. Oyedele O. A. Sulyok M. Somorin Y. M. (2019). Mycotoxins in poultry feed and feed ingredients in Nigeria. Mycotoxin Res. 35 (2), 149–155. 10.1007/s12550-018-0337-y 30484071 PMC6478637

[B2] Beloglazova N. V. Boevre M. D. Goryacheva I. Y. Werbrouck S. Guo Y. Saeger S. D. (2013). Immunochemical approach for zearalenone-4-glucoside determination. Talanta 106, 422–430. 10.1016/j.talanta.2013.01.020 23598147

[B3] Binder S. B. Schwartz-Zimmermann H. E. Varga E. Bichl G. Michlmayr H. Adam G. (2017). Metabolism of zearalenone and its major modified forms in pigs. Toxins (Basel) 9 (2), 56. 10.3390/toxins9020056 PMC5331435 28208710

[B4] Boevre M. D. Mavungu J. D. D. Landschoot S. Audenaert K. Eeckhout M. Maene P. (2012a). Natural occurrence of mycotoxins and their masked forms in food and feed products. World Mycotoxin J. 5 (3), 207–219. 10.3920/WMJ2012.1410

[B5] Boevre M. D. Mavungu J. D. D. Maene P. Audenaert K. Deforce D. Haesaert G. (2012b). Development and validation of an LC-MS/MS method for the simultaneous determination of deoxynivalenol, zearalenone, T-2-Toxin and some masked metabolites in different cereals and cereal-derived food. Food Addit. Contam. Part A 29 (5), 819–835. 10.1080/19440049.2012.656707 22369426

[B6] Caban M. Migowska N. Stepnowski P. Kwiatkowski M. Kumirska J. (2012). Matrix effects and recovery calculations in analyses of pharmaceuticals based on the determination of β-blockers and β-agonists in environmental samples. J. Chromatogr. A 1258, 117–127. 10.1016/j.chroma.2012.08.029 22935728

[B7] Chang H. Kim W. Park J. H. Kim D. Kim C. R. Chung S. (2017). The occurrence of zearalenone in South Korean feedstuffs between 2009 and 2016. Toxins (Basel) 9 (7), 223. 10.3390/toxins9070223 PMC5535170 28714869

[B8] Chen D. Cao X. Tao Y. Wu Q. Pan Y. Huang L. (2012). Development of a sensitive and robust liquid chromatography coupled with tandem mass spectrometry and a pressurized liquid extraction for the determination of aflatoxins and ochratoxin A in animal derived foods. J. Chromatogr. A 1253, 110–119. 10.1016/j.chroma.2012.06.095 22824215

[B9] Chilaka C. A. De B. M. Atanda O. O. De S. S. (2019). Fate of fusarium mycotoxins during processing of Nigerian traditional infant foods (ogi and soybean powder). Food Res. Int. 116, 408–418. 10.1016/j.foodres.2018.08.055 30716963

[B10] Cirlini M. Barilli A. Galaverna G. Michlmayr H. Adam G. Berthiller F. (2016). Study on the uptake and deglycosylation of the masked forms of zearalenone in human intestinal Caco-2 cells. Food Chem. Toxicol. 98, 232–239. 10.1016/j.fct.2016.11.003 27816555

[B11] Commission of the European Communities (2006). Commission Recommendation (EC) No 576/2006 of 17 August 2006 on the presence of deoxynivalenol, zearalenone, ochratoxin A, T-2 and HT-2 and fumonisins in products intended for animal feeding. Off. J. Eur. Union.. L229, 7–9.

[B12] Dall'Erta A. Cirlini M. Dall'Asta M. Rio D. D. Galaverna G. Dall'Asta C. (2013). Masked mycotoxins are efficiently hydrolyzed by human colonic microbiota releasing their aglycones. Chem. Res. Toxicol. 26 (3), 305–312. 10.1021/tx300438c 23347206

[B13] Danicke S. Winkler J. (2015). Invited review: Diagnosis of zearalenone (ZEN) exposure of farm animals and transfer of its residues into edible tissues (carry over). Food Chem. Toxicol. 84, 225–249. 10.1016/j.fct.2015.08.009 26277628

[B14] De B. M. Jacxsens L. Lachat C. Eeckhout M. Mavungu J. D. D. Audenaert K. (2013). Human exposure to mycotoxins and their masked forms through cereal-based foods in Belgium. Toxicol. Lett. 218 (3), 281–292. 10.1016/j.toxlet.2013.02.016 23454655

[B15] Dellafiora L. Galaverna G. Righi F. Cozzini P. Dall'Asta C. (2017). Assessing the hydrolytic fate of the masked mycotoxin zearalenone-14-glucoside - a warning light for the need to look at the "maskedome. Food Chem. Toxicol. 99 (1), 9–16. 10.1016/j.fct.2016.11.013 27856298

[B16] Dellafiora L. Perotti A. Galaverna G. Buschini A. Dall'Asta C. (2016). On the masked mycotoxin zearalenone-14-glucoside. does the mask truly hide? Toxicon 111, 139–142. 10.1016/j.toxicon.2016.01.053 26792714

[B17] EFSA Panel on Contaminants in the Food Chain (CONTAM) (2016). Appropriateness to set a group health-based guidance value for zearalenone and its modified forms. EFSA J. 14 (4), 4425. 10.2903/j.efsa.2016.4425

[B18] Eskola M. Kos G. Elliott C. T. Hajslova J. Mayar S. Krska R. (2020). Worldwide contamination of food-crops with mycotoxins: Validity of the widely cited ‘FAO estimate’ of 25%. Crit. Rev. Food Sci. Nutr. 60 (16), 2773–2789. 10.1080/10408398.2019.1658570 31478403

[B19] Gratz S. W. Dinesh R. Yoshinari T. Holtrop G. Richardson A. J. Duncan G. (2017). Masked trichothecene and zearalenone mycotoxins withstand digestion and absorption in the upper GI tract but are efficiently hydrolyzed by human gut microbiota *in vitro* . Mol. Nutr. Food Res. 61 (4), 1600680. 10.1002/mnfr.2016.00680 27921366

[B20] Gruber-Dorninger C. Jenkins T. Schatzmayr G. (2019). Global mycotoxin occurrence in feed: A ten-year survey. Toxins (Basel) 11 (7), 375. 10.3390/toxins11070375 PMC6669473 31252650

[B21] He Y. Ahmad D. Zhang X. Zhang Y. Wu L. Jiang P. (2018). Genome-wide analysis of family-1 UDP glycosyltransferases (UGT) and identification of UGT genes for FHB resistance in wheat (*Triticum aestivum L.*). BMC Plant Biol. 18 (1), 67. 10.1186/s12870-018-1286-5 29673318 PMC5909277

[B22] Isabell S. Karsten M. Gabriele E. Johann B. (2002). Occurrence of zearalenone-4-β-d- glucopyranoside in wheat. J. Agric. Food Chem. 50 (6), 1736–1738. 10.1021/jf010802t 11879067

[B23] Jeong Y. Schaffer A. Smith K. (2017). Equilibrium partitioning of organic compounds to OASIS HLB ® as a function of compound concentration, pH, temperature and salinity. Chemosphere 174, 297–305. 10.1016/j.chemosphere.2017.01.116 28183055

[B24] Jiang K. Huang Q. Fan K. Wu L. Nie D. Guo W. (2018). Reduced graphene oxide and gold nanoparticle composite-based solid-phase extraction coupled with ultra-high- performance liquid chromatography-tandem mass spectrometry for the determination of 9 mycotoxins in milk. Food Chem. 264, 218–225. 10.1016/j.foodchem.2018.05.041 29853368

[B25] Jin R. Li L. Guo L. Li W. Shen Q. A. (2017). A graphene tip coupled with liquid chromatography tandem mass spectrometry for the determination of four synthetic adulterants in slimming supplements. Food Chem. 224, 329–334. 10.1016/j.foodchem.2016.12.091 28159275

[B26] Kemboi D. C. Ochieng P. E. Antonissen G. Croubels G. Scippo M. L. Okoth S. (2020). Multi-mycotoxin occurrence in dairy cattle and poultry feeds and feed ingredients from Machakos Town, Kenya. Toxins (Basel) 12 (12), 762. 10.3390/toxins12120762 PMC7761711 33287105

[B27] Knutsen H. K. Alexander J. Barregard L. Bignami M. Bruschweiler B. Ceccatelli S. (2017). Risks for animal health related to the presence of zearalenone and its modified forms in feed. EFSA J. 15 (7), e04851. 10.2903/j.efsa.2017.4851 32625539 PMC7009830

[B28] Kosicki R. Błajet-Kosicka A. Grajewski J. Twarużek M. (2016). Multiannual mycotoxin survey in feed materials and feedingstuffs. Anim. Feed Sci. Technol. 215, 165–180. 10.1016/j.anifeedsci.2016.03.012

[B29] Labuda R. Parich A. Berthiller F. Tancinova D. (2005). Incidence of trichothecenes and zearalenone in poultry feed mixtures from Slovakia. Int. J. Food Microbiol. 105 (1), 19–25. 10.1016/j.ijfoodmicro.2005.06.005 16046021

[B30] Liu J. Applegate T. (2020). Zearalenone (ZEN) in livestock and poultry: Dose, toxicokinetics, toxicity and estrogenicity. Toxins (Basel) 12 (6), 377. 10.3390/toxins12060377 PMC7354539 32517357

[B31] Lorenz N. Danicke S. Edler L. Gottschalk C. Lassek E. Marko D. (2019). A critical evaluation of health risk assessment of modified mycotoxins with a special focus on zearalenone. Mycotoxin Res. 35 (1), 27–46. 10.1007/s12550-018-0328-z 30209771 PMC6331505

[B32] Ma R. Zhang L. Liu M. Su Y. T. Xie W. M. Zhang N. Y. (2018). Individual and combined occurrence of mycotoxins in feed ingredients and complete feeds in China. Toxins (Basel) 10 (3), 113. 10.3390/toxins10030113 PMC5869401 29518909

[B33] Majer-Baranyi K. Adanyi N. Szekacs A. (2021). Biosensors for deoxynivalenol and zearalenone determination in feed quality control. Toxins (Basel) 13 (7), 499. 10.3390/toxins13070499 34357971 PMC8310349

[B34] Mokubedi S. M. Phoku J. Z. Changwa R. N. Gbashi S. Njobeh P. B. (2019). Analysis of mycotoxins contamination in poultry feeds manufactured in selected provinces of South Africa using UHPLC-MS/MS. Toxins (Basel) 11 (8), 452. 10.3390/toxins11080452 PMC6722855 31382387

[B35] Munoz-Solano B. Gonzalez-Penas E. (2020). Mycotoxin determination in animal feed: An LC-FLD method for simultaneous quantification of aflatoxins, ochratoxins and zearelanone in this matrix. Toxins (Basel) 12 (6), 374. 10.3390/toxins12060374 PMC7354491 32516887

[B37] Palumbo R. Crisci A. Venancio A. Abrahantes J. C. Dorne J. L. Battilani P. (2020). Occurrence and co-occurrence of mycotoxins in cereal-based feed and food. Microorganisms 8 (1), 74. 10.3390/microorganisms8010074 PMC7023405 31947721

[B38] Pereira V. L. Fernandes J. O. Cunha S. C. (2014). Mycotoxins in cereals and related foodstuffs: A review on occurrence and recent methods of analysis. Trends Food Sci. Technol. 36 (2), 96–136. 10.1016/j.tifs.2014.01.005

[B39] Purohit T. J. Wu Z. Hanning S. M. (2020). Simple and reliable extraction and a validated high performance liquid chromatographic assay for quantification of amoxicillin from plasma. J. Chromatogr. A 1611, 460611. 10.1016/j.chroma.2019.460611 31627968

[B40] Ran R. Wang C. Han Z. Wu A. Zhang D. Shi J. (2013). Determination of deoxynivalenol (DON) and its derivatives: Current status of analytical methods. Food control. 34 (1), 138–148. 10.1016/j.foodcont.2013.04.026

[B41] Rausch A. K. Brockmeyer R. Schwerdtle T. (2020). Development and validation of a QuEChERS-based liquid chromatography tandem mass spectrometry multi-method for the determination of 38 native and modified mycotoxins in cereals. J. Agric. Food Chem. 68 (16), 4657–4669. 10.1021/acs.jafc.9b07491 32216338

[B42] Romera D. Mateo E. M. Mateo-Castro R. Gomez J. V. Gimeno-Adelantado J. V. Jimenez M. (2018). Determination of multiple mycotoxins in feedstuffs by combined use of UPLC-MS/MS and UPLC-QTOF-MS. Food Chem. 267 (30), 140–148. 10.1016/j.foodchem.2017.11.040 29934148

[B43] Ropejko K. Twaruzek M. (2021). Zearalenone and its metabolites-general overview, occurrence, and toxicity. Toxins (Basel) 13 (1), 35. 10.3390/toxins13010035 33418872 PMC7825134

[B44] Rubert J. Dzuman Z. Vaclavikova M. Zachariasova M. Soler C. Hajslova J. (2012). Analysis of mycotoxins in barley using ultra high liquid chromatography high resolution mass spectrometry: Comparison of efficiency and efficacy of different extraction procedures. Talanta 99, 712–719. 10.1016/j.talanta.2012.07.010 22967615

[B45] Santos P. C. Sara C. C. Fernandes J. O. (2019). Prevalent mycotoxins in animal feed: Occurrence and analytical methods. Toxins (Basel) 11 (5), 290. 10.3390/toxins11050290 PMC6563184 31121952

[B46] Tanveer Z. I. Huang Q. Liu L. Jiang K. Nie D. Pan H. (2020). Reduced graphene oxide-zinc oxide nanocomposite as dispersive solid-phase extraction sorbent for simultaneous enrichment and purification of multiple mycotoxins in Coptidis rhizoma (Huanglian) and analysis by liquid chromatography tandem mass spectrometry. J. Chromatogr. A 1630, 461515. 10.1016/j.chroma.2020.461515 32911177

[B47] Thieu N. Q. Ogle B. Pettersson H. (2008). Screening of aflatoxins and zearalenone in feedstuffs and complete feeds for pigs in Southern Vietnam. Trop. Anim. Health Prod. 40 (1), 77–83. 10.1007/s11250-007-9056-7 18551782

[B48] Vendl O. Berthiller F. Crews C. Krska R. (2009). Simultaneous determination of deoxynivalenol, zearalenone, and their major masked metabolites in cereal-based food by LC-MS-MS. Anal. Bioanal. Chem. 395 (5), 1347–1354. 10.1007/s00216-009-2873-y 19572123

[B49] Wen L. Liu L. Wang X. Wang M. L. Lin J. M. Zhao R. S. (2020). Spherical mesoporous covalent organic framework as a solid-phase extraction adsorbent for the ultrasensitive determination of sulfonamides in food and water samples by liquid chromatography-tandem mass spectrometry. J. Chromatogr. A 1625, 461275. 10.1016/j.chroma.2020.461275 32709327

[B50] Yang C. Song G. Lim W. (2020). Effects of mycotoxin-contaminated feed on farm animals. J. Hazard. Mat. 389 (5), 122087. 10.1016/j.jhazmat.2020.122087 32004836

[B51] Yang S. Zhang H. Zhang J. Li Y. Jin Y. Zhang S. (2018). Deglucosylation of zearalenone-14-glucoside in animals and human liver leads to underestimation of exposure to zearalenone in humans. Arch. Toxicol. 92 (9), 2779–2791. 10.1007/s00204-018-2267-z 30019167

[B52] Zhang Z. Nie D. Fan K. Yang J. Guo W. Meng J. (2020). A systematic review of plant-conjugated masked mycotoxins: Occurrence, toxicology, and metabolism. Crit. Rev. Food Sci. Nutr. 60 (9), 1523–1537. 10.1080/10408398.2019.1578944 30806521

[B53] Zhao L. Zhang L. Xu Z. Liu X. Chen L. Dai J. (2021). Occurrence of aflatoxin B1, deoxynivalenol and zearalenone in feeds in China during 2018-2020. J. Anim. Sci. Biotechnol. 12 (1), 74. 10.1186/s40104-021-00603-0 34243805 PMC8272344

[B54] Zinedine A. Soriano J. M. Molto J. C. Manes J. (2007). Review on the toxicity, occurrence, metabolism, detoxification, regulations and intake of zearalenone: An oestrogenic mycotoxin. Food Chem. Toxicol. 45 (1), 1–18. 10.1016/j.fct.2006.07.030 17045381

