# Supplementary material for: Development of a reliable UHPLC-MS/MS method for simultaneous determination of zearalenone and zearalenone-14-glucoside in various feed products
Source: Front Chem. 2022 Aug 10;10:955266. doi: 10.3389/fchem.2022.955266 (PMC9399508; doi:10.3389/fchem.2022.955266)
Supplement: Supplementary file 1 [file DataSheet1.docx]

Supplementary Material

#
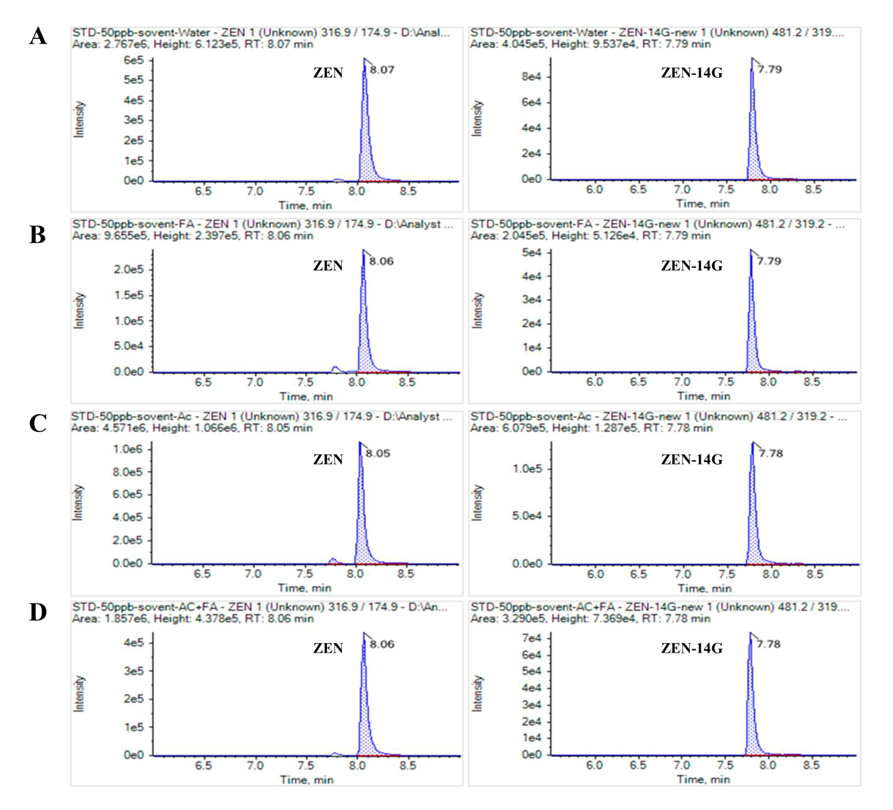


**Supplementary Figure 1.** Comparison of separation and ionization efficiencies of zearalenone (ZEN) and zearalenone-14-glucoside (ZEN-14G) among four candidate mobile phases. (A) methanol-water, (B) methanol-water containing 0.1% formic acid, (C) methanol-water containing 5 mmol L^–1^ ammonium acetate and (D) methanol-water containing 0.1% formic acid and 5 mmol L^–1^ ammonium acetate.


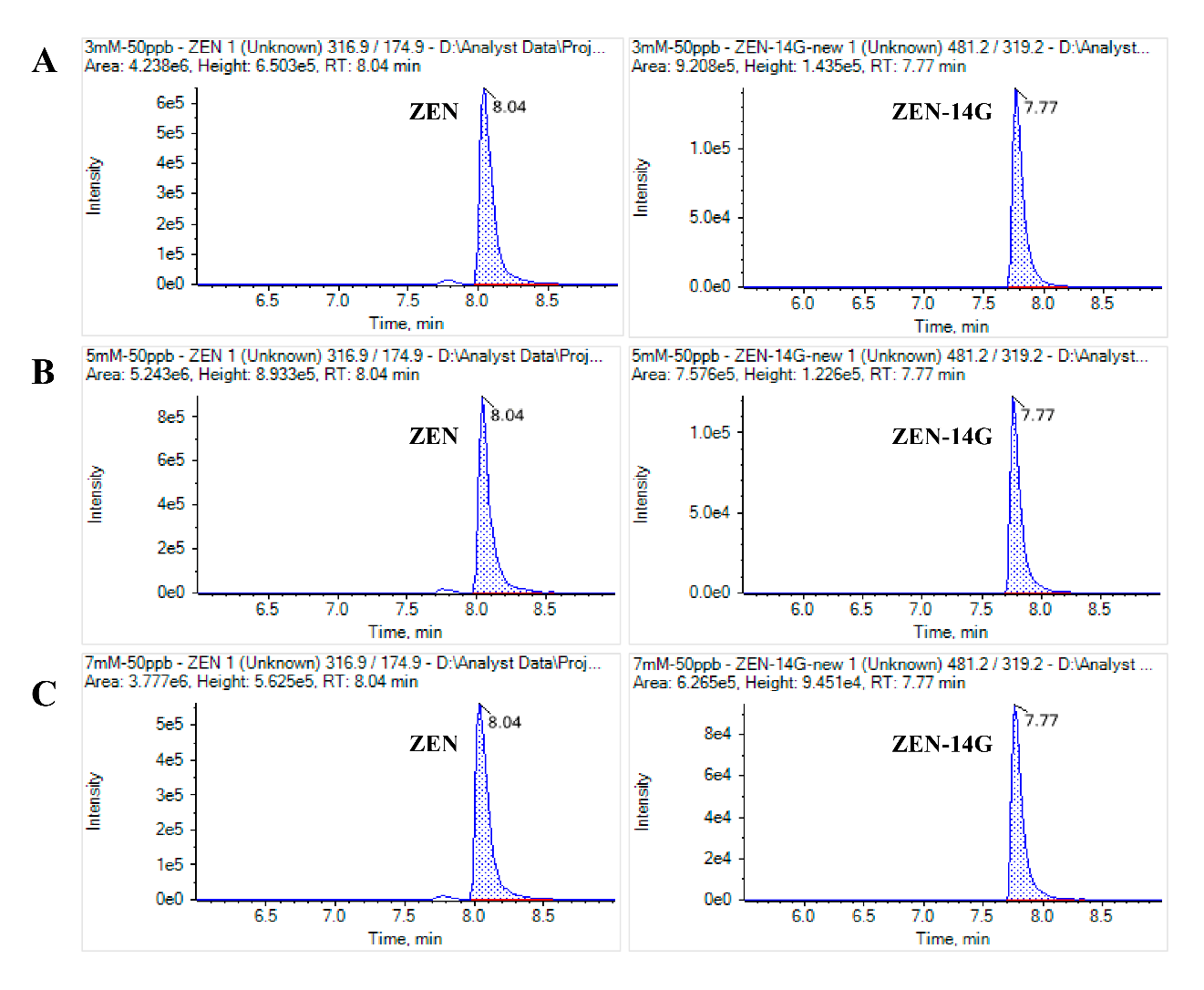


**Supplementary Figure 2.** Comparison of separation and ionization efficiencies of zearalenone (ZEN) and zearalenone-14-glucoside (ZEN-14G) among different percentages of ammonium acetate. (A) methanol-water containing 3 mmol L^-1^ ammonium acetate, (B) methanol-water containing 5 mmol L^-1^ ammonium acetate and (C) methanol-water containing 7 mmol L^-1^ ammonium acetate.

**
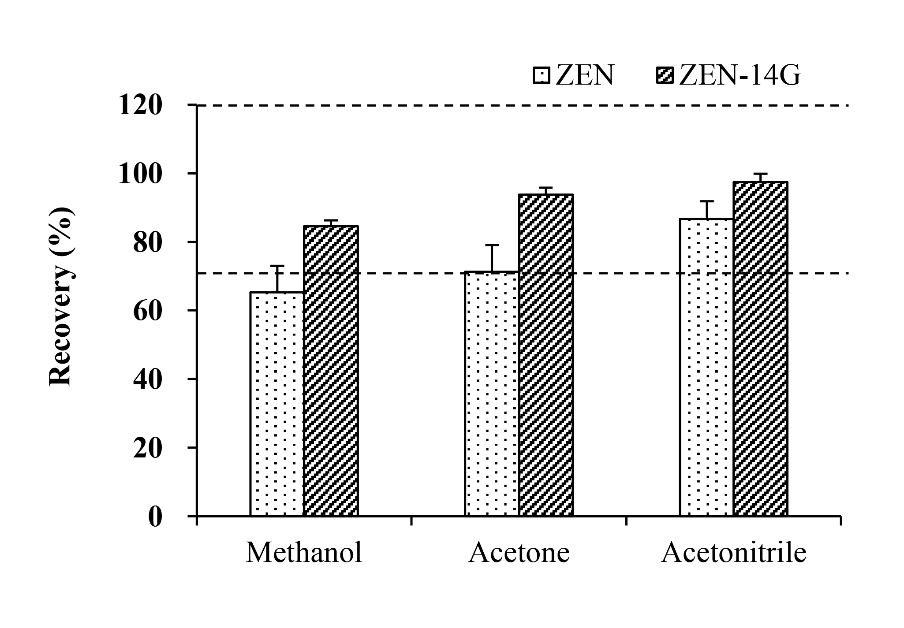
**

**Supplementary Figure 3.** Comparison of separation and ionization efficiencies of zearalenone (ZEN) and zearalenone-14-glucoside (ZEN-14G) among methanol, acetone and acetonitrile.


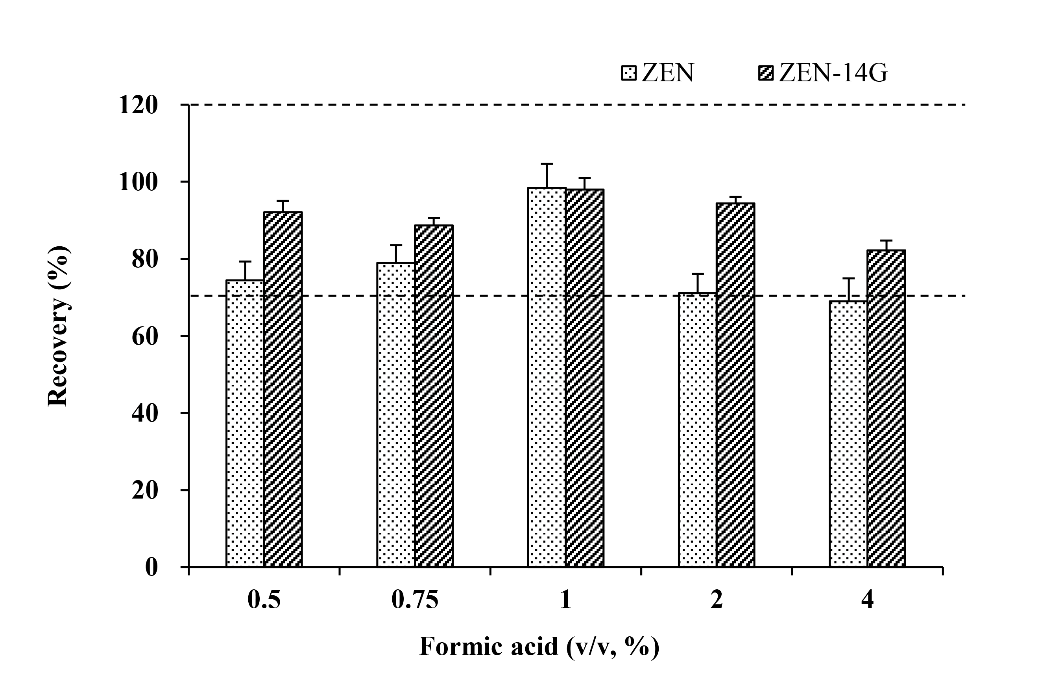


**Supplementary Figure 4.** Comparison of extraction efficiencies of zearalenone (ZEN) and zearalenone-14-glucoside (ZEN-14G) among different percentages of formic acid.


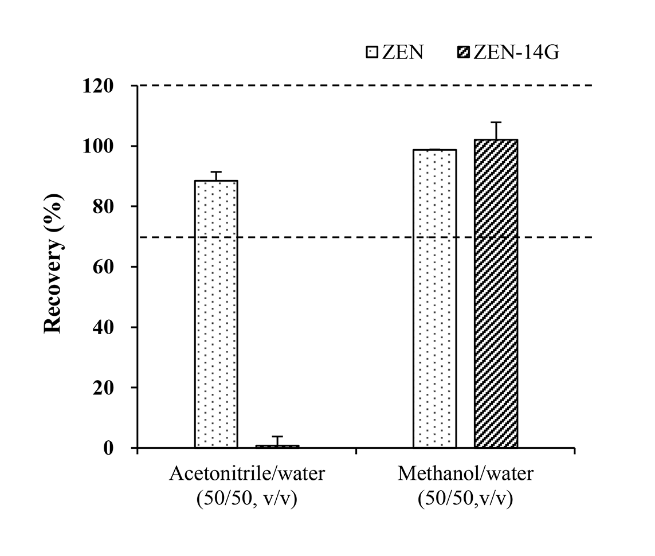


**Supplementary Figure 5.** Comparison of the performance for zearalenone (ZEN) and zearalenone-14-glucoside (ZEN-14G) with 50% of acetonitrile or methanol in washing solvents.


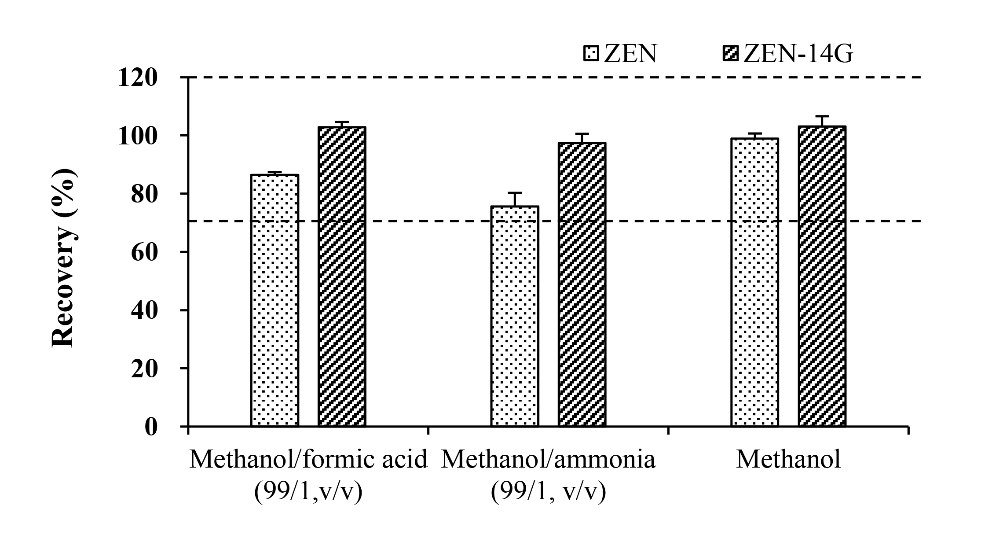


**Supplementary Figure 6.** Comparison of elution efficiencies for zearalenone (ZEN) and zearalenone-14-glucoside (ZEN-14G) by different solvents.

**Supplementary Figure 7.** The standard and the matrix-matched calibration curves spiked with zearalenone (ZEN) and zearalenone-14-glucoside (ZEN-14G). (A) calibration curve for ZEN in formula feed, (B) calibration curve for ZEN-14G in formula feed, (C) calibration curve for ZEN in concentrated feed, (D) calibration curve for ZEN-14G in concentrated feed, (E) calibration curve for ZEN in premixed feed, (F) calibration curve for ZEN-14G in premixed feed, (G) calibration curve for ZEN in standard solution and (H) calibration curve for ZEN-14G in standard solution.

**Supplementary Table 1.**

The molecular weights, molecular formulas, and chemical structures of zearalenone (ZEN) and zearalenone-14-glucoside (ZEN-14G).

| **Mycotoxins** | **Molecular Weight** | **Molecular Formula** | **Chemical Structure** |
| --- | --- | --- | --- |
| ZEN | 318.36 | C_18_H_22_O_5_ | 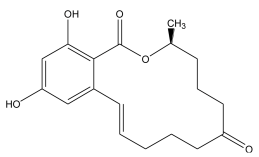 |
| ZEN-14G | 480.56 | C_24_H_32_O_10_ | 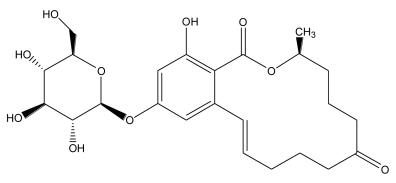 |

**Supplementary Table 2.**

Contamination levels of zearalenone (ZEN) and zearalenone-14-glucoside (ZEN-14G) in formula feed, concentrated feed and premixed feed samples (μg kg^-1^).

| **Formula Feed** | | | **Concentrated Feed** | | | **Premixed Feed** | | |
| --- | --- | --- | --- | --- | --- | --- | --- | --- |
| **Samples** | **ZEN** | **ZEN-14G** | **Samples** | **ZEN** | **ZEN-14G** | **Samples** | **ZEN** | **ZEN-14G** |
| **1** | 6.67 | - | **1** | 26.51 | - | **1** | 121.44 | - |
| **2** | 25.86 | - | **2** | 5.55 | - | **2** | 20.63 | - |
| **3** | 3.97 | - | **3** | - | - | **3** | 38.82 | 12.10 |
| **4** | 23.93 | - | **4** | 10.51 | 6.99 | **4** | 3.00 | - |
| **5** | 10.36 | 6.12 | **5** | - | - | **5** | 9.70 | 0.89 |
| **6** | 4.32 | 6.43 | **6** | - | - | **6** | 38.96 | 5.90 |
| **7** | 2.08 | - | **7** | 44.28 | - | **7** | 19.72 | 1.05 |
| **8** | 11.80 | 7.80 | **8** | 0.63 | - | **8** | 89.79 | - |
| **9** | 22.35 | 6.53 | **9** | 62.30 | 5.76 | **9** | 194.22 | - |
| **10** | 7.19 | 8.85 | **10** | 38.90 | - | **10** | 48.57 | 15.31 |
| **11** | 1.71 | 8.47 | **11** | 28.07 | - | **11** | 119.66 | 10.63 |
| **12** | 4.25 | 8.82 | **12** | 9.87 | 9.26 | **12** | 49.62 | 1.16 |
| **13** | 24.62 | - | **13** | 28.00 | - | **13** | 32.05 | 4.28 |
| **14** | 5.22 | - | **14** | 0.71 | 5.28 | **14** | 29.76 | - |
| **15** | 6.08 | - |  |  |  | **15** | - | - |
| **16** | 7.26 | - |  |  |  | **16** | 18.64 | 2.52 |
| **17** | - | 6.40 |  |  |  | **17** | 56.13 | 1.75 |
| **18** | 381.19 | - |  |  |  | 18 | - | - |
| **19** | 615.24 | - |  |  |  |  |  |  |
| **20** | - | - |  |  |  |  |  |  |
| **21** | 29.45 | - |  |  |  |  |  |  |
| **22** | 29.21 | - |  |  |  |  |  |  |
| **23** | 14.26 | - |  |  |  |  |  |  |
| **24** | 13.08 | - |  |  |  |  |  |  |
| **25** | - | - |  |  |  |  |  |  |
| **26** | - | - |  |  |  |  |  |  |
| **27** | - | - |  |  |  |  |  |  |
| **28** | 17.99 | - |  |  |  |  |  |  |

“-“: Below the limit of quantification.

**Supplementary Table 3.**

Regulations set in the European Union (EU) and China for zearalenone (ZEN) in different feedingstuffs [1, 2].

| **Item** | **Species** | | **Feeds** | **ZEN (μg kg^-1^)** |
| --- | --- | --- | --- | --- |
| EU | Swine | Piglets and gilts  (young sows) | Complementary and complete feedingstuffs | 100 |
|  |  | Sows and fattening pigs | Complementary and complete feedingstuffs | 250 |
|  | Ruminants |  | Complementary and complete feedingstuffs | 500 |
|  | Poultry |  | - | - |
| China | Swine | Piglets | Complete feed | 150 |
|  |  | Young sows | Complete feed | 100 |
|  |  | Other pigs | Complete feed | 250 |
|  | Others |  | Complete feed | 500 |

1. Commission, E., Commission recommendation of 17 August 2006 on the presence of deoxynivalenol, zearalenone, ochratoxin A, T-2 and HT-2 and fumonisins in products intended for animal feeding. 2006.

2. Hygienical Standard for Feeds; GB/T 13078-2017; National Criterion of China: Beijing, China, 2017.
